# Sharpening our public health lens: advancing im/migrant health equity during COVID-19 and beyond

**DOI:** 10.1186/s12939-021-01399-1

**Published:** 2021-02-08

**Authors:** Stefanie Machado, Shira Goldenberg

**Affiliations:** 1 Centre for Gender & Sexual Health Equity, c/o St Paul’s Hospital, 1081 Burrard, BC V6Z 1Y6 Vancouver, Canada; 2 Faculty of Health Sciences, Simon Fraser University, BC Burnaby, Canada; 3 Division of Infectious Diseases & Global Public Health, University of California San Diego, CA San Diego, USA

**Keywords:** Public health, Immigrant health, Health equity, COVID-19

## Abstract

**Background:**

Differential impacts of the COVID-19 pandemic have brought deeply rooted inequities to the forefront, where increasing evidence has shown that racialized immigrant and migrant (im/migrant) populations face a disproportionate burden of COVID-19. Im/migrant communities may be worst affected by lockdowns and restrictive measures, face less opportunity to physically distance or stay home sick within ‘essential’ jobs, and experience severe barriers to healthcare. Insufficient attention to experiences of racialized im/migrants in current pandemic responses globally highlights an urgent need to more fulsomely address unmet health needs through an anti-racist, equity-oriented lens. This commentary aims to highlight the need for public health and clinical training, research, and policy to thoughtfully prioritize im/migrant health equity during and beyond the COVID-19 pandemic.

**Main text:**

Global pandemic responses have neglected im/migrants by continuing to ignore or insufficiently address inequities, exacerbating COVID transmission, xenophobia, and occupational injustice. Deaths, illness, stress, and other negative outcomes of the overlapping epidemics of COVID-19 and structural racism disproportionately borne by racialized im/migrants suggest the urgent need for action. As evidence mounts about how im/migrants have been left behind in times of crises, we need enhanced focus on health equity within COVID-19 research and interventions, including research that examines and pursues structural interventions necessary to mitigate these impacts, and that identifies patterns and harms of xenophobic policy, structural racism, and white supremacy in shaping im/migrant health outcomes. We must also strengthen anti-racist and equity-oriented curriculum within health education, and ensure sufficient attention to the needs of im/migrant communities within public health, clinical, and research training.

**Conclusion:**

The COVID-19 pandemic has exacerbated and rendered more visible the deeply rooted health and social inequities faced by racialized im/migrants across diverse settings. We argue for a greater emphasis on equity-focused and anti-racist im/migrant health research, interventions, and training. Policymakers and practitioners must ensure that healthcare policies and practices do not exacerbate inequities, and instead meaningfully address unmet needs of communities, including racialized im/migrants. Ethical and respectful community engagement, commitment and collaboration with global, national, and local communities, policymakers, academics, and educators, as well as accountability across sectors, is critical.

## Background

The COVID-19 pandemic has swept the globe, but its differential impacts among racialized and marginalized immigrant populations have brought deeply rooted inequities to the forefront. Racialization continues to ensure that the identities of immigrants within social structures and institutions are defined by their race, leading to severe inequities among immigrants of colour [1]. Increasing evidence has shown that outbreaks in settings such as farms, meat processing plants and residential and long-term care centres, where racialized im/migrants are overrepresented, has perpetuated a disproportionate burden of COVID-19 transmission for these groups [2, 3]. We use the term “im/migrant” to include all immigrants and migrants, including refugees, asylum seekers, and undocumented persons. Lower income im/migrant communities may also be worst affected by lockdowns and restrictive measures, face less opportunity to physically distance or stay home sick within ‘essential’ jobs, and are known to face severe barriers to healthcare [3]. The insufficient attention to experiences of racialized im/migrants in policy responses during COVID-19, including government benefits, occupational environments, and public health, highlights an urgent need to more fulsomely address these unmet needs. Given the current need for rapid data in order to inform pandemic responses and growing evidence of inequities in COVID-19 transmission and impacts among racialized im/migrant communities, this commentary highlights the need for public health training, research, and policy to better prioritize and address inequities among racialized im/migrants.

### COVID-19 has further exposed inequitable health outcomes faced by racialized im/migrants globally

Global pandemic responses have neglected im/migrants by continuing to ignore or at best, insufficiently address inequities, exacerbating COVID transmission, xenophobia, racism, and occupational injustice [4]. Xenophobic immigration policies demonstrate the ways in which communities without or who are seeking immigration status find themselves deemed ‘unworthy’ of protection and basic human rights. Deaths, illness, stress, and other negative consequences of overlapping issues of COVID-19 and precarious im/migration status highlight the tangible, life-threatening manifestation of these inequities, perpetuating structural racism. Worse yet, dominant public health interventions (e.g., sweeping ‘lockdowns’) may reinforce inequities by privileging more advantaged groups – for example, those who are able to work remotely, access private childcare during school closures, and engage in virtual services [5] – while failing to sufficiently adopt strategies that support more marginalized populations.

These inequities manifest across diverse settings. Despite portrayals of Canada as a setting of universal healthcare and inclusion, racialized immigrants with and without status face stark health and social inequities prior to and during COVID-19, including racial profiling by police, inabilities to meet basic needs, and barriers to healthcare [3]. Im/migrants working in care centres, healthcare settings, and farms are heavily overrepresented among COVID-19 cases. Migrant farmworkers in particular have reported coercion into unsafe work environments and threats of deportation, alongside other rights violations (e.g., termination based on country of origin) [2]. Lockdowns in India have forced 100 million migrant workers into unemployment, where many have fled to home communities by foot and died from hunger and exhaustion [6]. Despite being called upon by the United Nations to protect migrants’ rights, the government’s inaction continues to exacerbate poverty, police brutality and COVID-19 stigma among workers [6]. Germany’s recent global health strategy effectively excluded refugees and asylum seekers from their pandemic response, contributing to several outbreaks among im/migrant populations [7]. Meanwhile, in the United States, Customs and Border Patrol continues to discriminately deport asylum seekers and incarcerate im/migrant children and families in crowded and unsafe detention centres, in direct violation of national and global policies and human rights standards [8]. These examples highlight the urgent need to shift the gaze of public health to prioritize and address the deep-rooted and alarming crisis currently being faced by im/migrant communities across diverse high, middle, and low-income contexts during COVID-19.

### We need enhanced focus on im/migrant health equity within COVID-19 research and interventions

As evidence mounts about how racialized immigrants have been left behind in times of crises, there is a need to sharpen public health decision-making and evidence with intentional considerations of equity and social justice. Alongside crucially needed biomedical work including COVID-19 testing and vaccine development, there is a need to address deep inequities being produced and exacerbated by the COVID-19 pandemic by examining and pursuing structural interventions that are necessary to mitigate these impacts [5]. In the context of im/migrant health equity, research identifying patterns and harms of xenophobic policy, structural racism, and white supremacy [9] in shaping im/migrant health outcomes during and beyond COVID-19 is needed. Research and structural interventions must address equity and structural racism, and be sufficiently tailored to pandemic phases and community contexts. Areas of urgently needed focus may include occupational protections (e.g., sick pay, ability to physically distance), healthcare and social protection schemes available to all residents regardless of im/migration status, training for public health practitioners and clinicians focused on anti-racist, culturally tailored approaches, and decarceration in jails, prisons, and immigration detention.

Finally, there is a need to strengthen anti-racist and equity-oriented curriculum within health education, and ensure sufficient attention to the needs of im/migrant communities within public health, clinical, and research training. This includes addressing the severe structural challenges faced by international students across settings during the pandemic; [9] during COVID-19, this has involved learning across time zones, experiencing family separation, navigating financial difficulties, and facing racism, xenophobia, and fear associated with im/migration status, [10] as demonstrated by the recent U.S. policy proposal to force international students to return to their countries of origin for programmes shifted solely online.

## Conclusions

The COVID-19 pandemic has exacerbated and rendered more visible the deeply rooted health and social inequities faced by racialized im/migrants across diverse settings. In this commentary, we argue for a greater emphasis on equity-focused and anti-racist im/migrant health research, interventions, and training. Policymakers and practitioners have a responsibility to ensure that healthcare policies and practices do not exacerbate inequities, and instead meaningfully address unmet needs of communities, including racialized im/migrants. Ethical and respectful community engagement is critical for achieving this. To fulsomely advance equity-focused research and interventions, deep commitment and collaboration with global, national, and local communities, policymakers, academics, and educators, as well as accountability across sectors, is needed.

## Data Availability

Not applicable.
